# Prediction of gaps in atrial ablation lesion sets by late gadolinium enhancement magnetic resonance imaging

**DOI:** 10.1186/1532-429X-15-S1-E95

**Published:** 2013-01-30

**Authors:** James Harrison, Nick Linton, Steven Williams, Rashed Karim, Kawal Rhode, Matthew Wright, Tobias Schaeffter, Reza Razavi, Mark O'Neill

**Affiliations:** 1 Division of Imaging Sciences & Biomedical Engineering, King's College London, London, UK; 2 Department of Cardiology, St Thomas' Hospital, London, UK

## Background

Arrhythmia recurrence following acutely successful catheter ablation for atrial fibrillation (AF) is associated with recovery of conduction across previously complete circumferential or left atrial (LA) linear lesions. Late gadolinium enhancement (LGE) magnetic resonance imaging (MRI) may be able to provide a non-invasive means to identify gaps in ablation lesions. This study assessed this prospectively in patients undergoing repeat LA catheter ablation.

## Methods

Eleven patients who had previously undergone one or more LA catheter ablation procedures for AF, and who represented with either paroxysmal AF (n=4) or atrial tachycardia (AT) (n=7) underwent LGE MRI (Figure) 2-3 weeks prior to repeat catheter ablation. The scans were not analysed before repeat ablation and the procedure was performed without using any of the anatomical or scar information from the LGE MRI.

**Figure 1:**
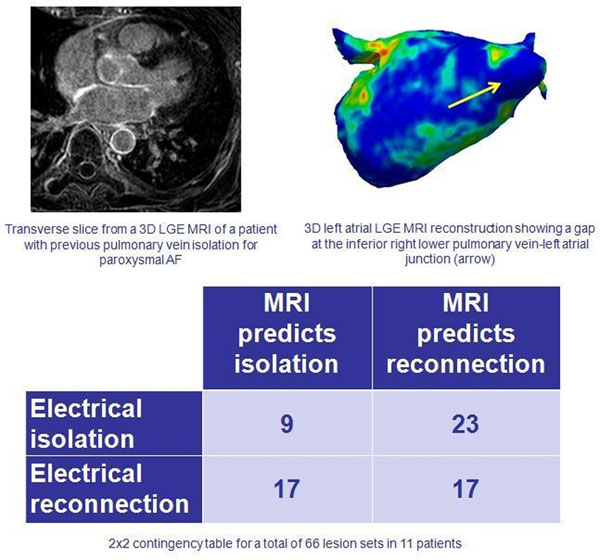


After ablation, three expert blinded reviewers (who were not operators in the repeat procedure) independently scored 3D left atrial (LA) LGE MRI reconstructions (Figure 1) for the presence or absence of gaps at the pulmonary vein-LA junction and across the roof line and mitral line (making a total of 66 lesion sets). Electrical integrity of these lesion sets had been assessed at the time of repeat catheter ablation.

## Results

From the LGE MRI scans, interobserver agreement for detection of any gap was 95%. The positive predictive value of LGE MRI for gap detection was 35%, whilst the negative predictive value was 43%. Specificity and sensitivity were 50% and 28% respectively.

## Conclusions

There was a very high degree of agreement between the three reviewers as to the presence or absence of a gap on LGE MRI. However, these gaps detected by LGE MRI were not validated by invasive electrical assessment at the time of repeat catheter ablation.

## Funding

British Heart Foundation Clinical Research Training Fellowship.

